# Modified pedicle screw-rod fixation versus anterior pelvic external fixation for the management of anterior pelvic ring fractures: a comparative study

**DOI:** 10.1186/s13018-017-0688-7

**Published:** 2017-12-01

**Authors:** Chun Bi, Qiugen Wang, Jianhong Wu, Feng Zhou, Fei Zhang, Haipeng Liang, Fei Lyu, Jiandong Wang

**Affiliations:** Department of Orthopedics Trauma, Trauma Center, Shanghai General Hospital, School of Medicine, Shanghai Jiao Tong University, 650 Xin Songjiang Road, Shanghai, 201620 People’s Republic of China

**Keywords:** Pelvic fracture, Anterior ring, Modified pedicle screw-rod fixation, External fixation

## Abstract

**Background:**

Anterior pelvic ring fracture, as high-energy trauma, needs to be effectively treated. The purpose of the current study was to evaluate the clinical applications of modified pedicle screw-rod fixation and anterior pelvic external fixation for the treatment of anterior pelvic ring fracture.

**Methods:**

Either modified pedicle screw-rod fixation (modified PSRF group, *N* = 21) or anterior pelvic external fixation (APEF group, *N* = 22) was performed to 43 patients, with or without fixation of posterior ring. Clinical outcomes were evaluated via Majeed scores. Relevant clinical evaluation indicators including operation time, intraoperative blood loss, hospitalization duration, and complications were compared between these two groups.

**Results:**

The operation time in APEF group was significantly less than that in modified PSRF group (*P* < 0.0001). No significant difference with respect to intraoperative blood loss and hospitalization duration between the two groups was shown (*P* = 0.51 and *P* = 0.33, respectively). Six patients developed surgical site infection in APEF group. Three patients experienced loss of fixation, and two patients experienced loosening of fixator in APEF group. Temporary lateral femoral cutaneous nerve irritation occurred in three patients in modified PSRF group while two patients in APEF group. One patient experienced femoral nerve palsy in modified PSRF group. Fractures of all patients healed well eventually. No statistical difference regarding Majeed evaluation scores was found between two groups.

**Conclusions:**

Application of both modified PSRF and APEF could provide similar satisfactory clinical outcomes for anterior pelvic ring fracture. Modified PSRF, a minimally invasive technique with the advantages of internal fixation, could be performed as an alternative method for instable pelvic fractures.

**Trial registration:**

Research Registry UIN: researchregistry2776.

## Background

Accounting for only 6% of all fractures, the high-energy pelvic ring fractures often lead to serious consequences with high mortality and morbidity [1]. While a variety of treating methods have been employed, successful management of unstable pelvic fractures remains a challenge to orthopedic surgeons [2]. As a quick and easy fixation method, anterior pelvic external fixation (APEF) can stabilize the disrupted pelvic ring rapidly. Its application has been proved to efficiently reduce the mortality and morbidity rates with less operation time as well as the blood loss compared with open fixation by plate [3–6]. Unfortunately, its application is not without complications. Tract infection, fixator loosen, restricted daily activities, and the skin problem caused by fixator are the main concerns. Previous studies have shown that the incidence of these complications can be as high as 60% [7–10].

Recently, minimally invasive techniques, with the potential merits of reduced blood loss, faster fixation, and less soft tissue injuries, have been widely recommended for anterior pelvic fixation [11–15]. A novel method of these techniques is to perform two pedicle screws fixed into the ilium and use a curved rod for connection [14, 15]. We modified this technique in clinical practice by adding another pedicle screw in the region of pubis, defined as modified pedicle screw-rod fixation (modified PSRF), to improve the fixation strength.

The current study aims to evaluate the clinical effects of modified PSRF and APEF for treating unstable anterior pelvic ring fractures. Shanghai General Hospital’s Ethics Committee reviewed and approved this retrospective study. Each participant signed the written informed consent. All procedures were performed in the light of the Declaration of Helsinki.

## Methods

Between September 2012 and November 2016, totally, 43 patients with unstable pelvic fractures underwent either minimal invasive pedicle screw-rod fixation or APEF, with or without posterior fixation. Patients with hemodynamic instability, serious osteoporosis, and open fractures with severely soft tissue defects were excluded.

According to Tile classification, there were 43 patients of type B (8 type B1, 19 type B2, and 16 type B3) (Table 1). These patients involved 21cases of traffic accidents, 12 cases of crushes, and 10 cases of fall from height. The choice of managements with either modified pedicle screw-rod fixation (*N* = 21, modified PSRF group) or APEF (*N* = 22, APEF group) was based on the level of injuries and experience of the orthopedic surgeons.

**Table 1:** Patient demographics

	Modified PSRF	APEF	*P* value
Gender (male: female)	12:9	12:10	0.86
Fracture type (B1:B2:B3)	5:9:7	3:10:9	0.43
Age (years)	37.85 ± 10.31	34.40 ± 9.42	0.27
Operation time (min)	53.90 ± 5.34	47.50 ± 4.00	< 0.0001
Intraoperative blood loss (ml)	33.60 ± 5.34	32.55 ± 4.21	0.51
Hospitalization duration (days)	8.95 ± 1.64	8.47 ± 1.52	0.33
Majeed evaluation score	83.29 ± 7.68	80.68 ± 9.11	0.32
Follow-up (months)	16.57 ± 2.11	16.31 ± 2.17	0.7
Additional posterior ring fixation (*n*)	21	7	N/A

*N/A* not available

Anteroposterior, inlet, and outlet pelvic radiographs were performed in all patients. To make further and better evaluation of the displaced fracture, 3-D computed tomography (CT) scans of the pelvis in all cases were taken preoperatively.

### Surgical procedures

Firstly, posterior pelvic ring was addressed as the priority of fixation in all cases to acquire the stabilization of the posterior pelvis. Due to the minimal damage and easy-operating of PSRF to the surrounding tissue, pedicle screws and titanium rod were used in all cases (21 cases) for posterior fixation in modified PSRF group. While in APEF group, the locking compression plate was performed to the patients with unstable posterior ring fractures (7 cases) due to the potential stimulation of locking compression plate to the local soft tissue. The anterior ring fixation was performed after the posterior pelvis being stabilized.

#### Modified PSRF

**Fig. 1:**
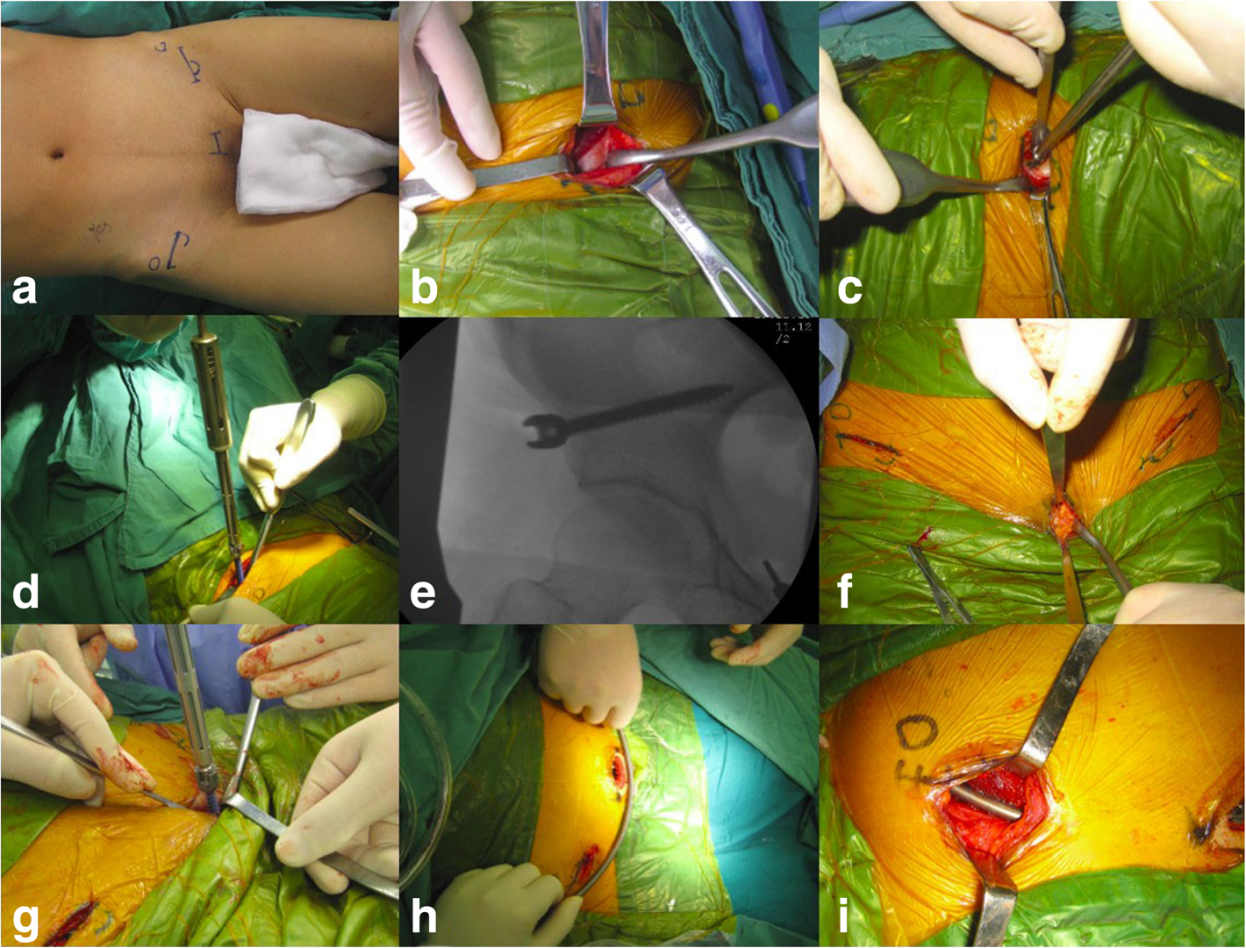
**a**–**i** The step-by-step illustrations in procedures of PSRF. **a** the site of anterior inferior iliac spine (AIIS) and the pubis symphysis including its centerline were marked. **b** the AIIS was explored after the blunt dissection was taken between the space of the sartorius and the iliopsoas followed by drawing the sartorius outward. **c** the starting point was selected at the lateral one-third side of the AIIS, and the bony corridor was created by the pedicle finder. **d**, **e** the pedicle screw with the diameter of 7 mm and the length of 80 mm was inserted with the suitable depth in the outward tilt angle of 30° as well as the backward tilt angle of 20°. **f** 2-cm incision was positioned over the pubic tubercle. **g** pedicle screw with the diameter of 6.5 mm and the length of 50 mm was placed in appropriate depth under the X-ray fluoroscopy. **h** three pedicle screws were fixed and then a titanium rod with 6 mm diameter was curved according to the shape of anterior ring. **i** long hemostat was used to make the corridor superficial to the fascia from the incision from bilateral AIIS to the pubic tubercle, then the titanium rod was placed through the corridor passing below the sartorius and the front of medial iliopsoas. And then, it was connected to these three pedicle screws head

The site of anterior inferior iliac spine (AIIS) and the pubis symphysis including its centerline were marked, respectively (Fig. 1a). A transverse incision with 4-cm length was made 2 cm below the AIIS. The AIIS was explored after the blunt dissection was taken between the space of the sartorius and the iliopsoas followed by drawing the sartorius outward (Fig. 1b). The starting point was selected at the lateral one-third side of the AIIS, and the bony corridor was created by the pedicle finder (Fig. 1c). After ensuring the corridor did not penetrate the ilium, the pedicle screw with the diameter of 7 mm and the length of 80 mm was inserted with the suitable depth in the outward tilt angle of 30° as well as the backward tilt angle of 20° (Fig. 1d, e). At the site of AIIS in the contralateral pelvis, we performed the same procedure.

**Fig. 2:**
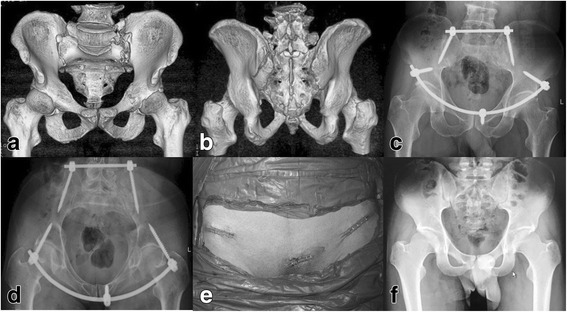
A 57-year-old male patient with anterior and posterior pelvic ring fracture because of a crushing injury. **a**, **b** Preoperative 3-D CT image showing the anterior pelvic ring fracture. **c**, **d** Postoperative X-ray film showing the satisfactory reduction with modified pedicle screw-rod fixation (modified PSRF). **e** The postoperative incision. **f** X-ray film showing the healed fracture at postoperative 8 months

A 2-cm incision was positioned over the pubic tubercle (Fig. 1f). A pedicle screw with the diameter of 6.5 mm and the length of 50 mm was placed in appropriate depth under the X-ray fluoroscopy (Fig. 1g). Totally, three pedicle screws were fixed and then a titanium rod with 6 mm diameter was curved according to the shape of anterior ring (Fig. 1h). A long hemostat was used to make the corridor superficial to the fascia from the incision from bilateral AIIS to the pubic tubercle, then the titanium rod was placed through the corridor passing below the sartorius and the front of medial iliopsoas. And then, it was connected to these three pedicle screws head (Fig. 1i). After ensuring and adjusting the rod to the right place, the caps of these pedicle screws were tightened by the screwdriver. The incision was closed and coated with gauze after carefully cleaning layer by layer. A typical patient was shown in Fig. 2.

#### APEF

**Fig. 3:**
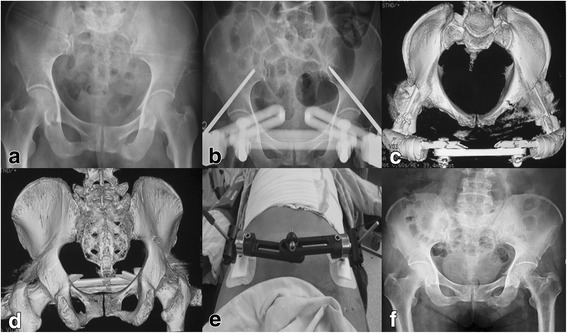
A 60-year-old female patient with anterior pelvic ring fracture due to a traffic accident. **a** Preoperative X-ray film showing the anterior pelvic ring fracture. **b**–**d** Postoperative X-ray film and 3-D CT showing the satisfactory reduction with anterior pelvic external fixation (APEF). **e** The postoperative incision. **f** X-ray film showing the healed fracture at postoperative 7 months

**Fig. 4:**
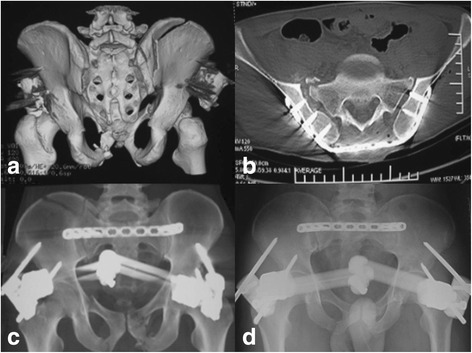
A 48-year-old male patient with anterior and posterior pelvic ring fracture due to a crushing injury. **a** 3-D CT image showing the anterior pelvic ring fracture. **b**, **c** Postoperative X-ray film showing the satisfactory reduction with anterior pelvic external fixation (APEF) and posterior pelvic fixation using locking compression plate (LCP). **d** X-ray film showing the healed fracture at postoperative 5 months

After being placed in supine position, the patient was managed by APEF formed by two-pin and two-bar complex. A 1-cm skin incision was made two-finger breaths of clearance below the anterior inferior iliac spine (AIIS). Soft tissue splitter was used outward to explore the AIIS. A 5-mm diameter hydroxylapatite coating pin was employed at the AIIS site on each side of the pelvis under the X-ray fluoroscopy. The pins were connected to the external fixation bars. After adjusting the length of the connecting bar, the pins were fixed. Then, the incision was closed and coated with gauze after carefully cleaning layer by layer. Typical patients with or without LCP posterior fixation were shown in Figs. 3 and 4, respectively.

### The postoperative rehabilitation

After being sent to the orthopedic ward, all patients were maintained on a non-weight-bearing status on the affected side for 24 h, postoperatively.

#### Modified PSRF

After acute pain period, the body positions of patients were changed to complete sitting position. Then, as long as the pain could be tolerated, the patients were encouraged to take active and positive exercises 3 days after operations. From 3 days to 2 weeks postoperatively, the crutch-assisted walking was performed by the patients, with affected side partial weight-bearing and unaffected side full weight-bearing. After 2 weeks, the affected side weight-bearing was increased gradually. Full weight bearing of all patients was advocated at 6 weeks, postoperatively.

#### APEF

After acute pain period, the body positions of patients were changed to semi-sitting position. Then, as long as the pain could be tolerated, 3 days after operations, the patients were encouraged to take active and positive exercises. From 1 week postoperatively, the crutch-assisted walking was performed by the patients, with affected side partial weight-bearing and unaffected side full weight-bearing. After 3 weeks, the affected side weight-bearing was increased gradually. Full weight bearing of all patients except for type B3 fractures was advocated at 6 weeks, postoperatively. For patients of type B3 fractures with bilateral pubic fractures, the stretching of adductor due to weight bearing as well as the skin irritation of APEF could lead to pain and discomfort which results in these patients were reluctant to full weight bearing. Full weight bearing of these patients was started at 2 months after operations.

### Statistical analysis

By means of SPSS v. 19.0 software (Chicago, IL, USA), all data were analyzed. The collected data were presented as mean and SD. The Student *t* test was used to compare the data between two groups. *p* value of < 0.05 was considered statistically significant.

## Results

In the present study, there were totally 43 patients among them, 12 male and 9 female in the modified PSRF group as well as 12 male and 10 female in the APEF group. The characteristics of patients were presented in Table 1. In modified PSRF group, the patients’ average age was 37.9 years (range 22–56 years), while the average age in APEF group was 34.4 years (range 23–55 years).

### Relevant surgical evaluation indicators

Operation time in modified PSRF group ranged from 46 to 63 min with the mean time of 53.9 min. While in APEF group, the operation time ranged from 42 to 56 min and the mean time was 47.5 min. Significant difference was shown (*P* < 0.0001).

The mean intraoperative blood loss for modified PSRF and APEF was 33.6 ml (range 23–45 ml) and 32.6 ml (range 24–40 ml), respectively (*P* = 0.51). The two groups did not differ significantly in terms of hospitalization duration (*P* = 0.33).

### Follow-up

The mean follow-up time for modified PSRF group was 16.6 months (range 12–20 months) and for APEF group was 16.3 months (range 13–20 months). During the follow-up period, no delayed osseous union or nonunion was shown from the clinical physical examination and X-ray films for both two groups. Fractures of all patients healed well eventually. Majeed evaluation scores were performed 1 year postoperatively for both groups. In modified PSRF group, the results showed excellent in 10, good in 9, and fair in 2. The scores ranged from 68 to 94 (83.29 ± 7.68). In APEF group, the results were rated as excellent in 9, good in 8, and fair in 5, with the scores ranged from 64 to 93 (80.68 ± 9.11). No statistical difference regarding Majeed evaluation scores was found between the two groups.

### Complications

Six patients developed surgical site infection in APEF group. Three patients experienced loss of fixation, and two patients experienced loosening of fixator in APEF group. Temporary LFCN (lateral femoral cutaneous nerve) irritation occurred in three patients in modified PSRF group and two patients in APEF group. There was one patient who had femoral nerve palsy in modified PSRF group (Table 2).

**Table 2:** Complications of two groups

	Modified PSRF (*n*)	APEF (*n*)
Surgical site infection	0	6
Loss of fixation	0	3
Loosening of implants	0	2
LFCN irritation	3	2
Femoral nerve palsy	1	0

## Discussion

Although the main stability of pelvis is sustained by posterior ring, anterior ring, as a significant anatomical component formed by pubic symphysis, pubic ramus, pubic tubercle, and ventral ilium, provides 30% of the pelvic stability as well [16–18]. Thus, to acquire better reduction of unstable pelvic fracture, a combination of anterior and posterior fixation is needed, if necessary.

APEF, as a time-tested technique, has an outstanding advantage of rapid stabilization of the fractured pelvis with rotational, vertical as well as posterior instability [19]. The APEF is frequently performed to augment the pelvic stability. Its application has demonstrated to effectively reduce the mortality of the pelvic injuries [20]. However, treating technique using APEF is not without shortcomings. Surgical site infection, loss of fixation, loosening of the fixator, inconvenience to daily life, etc. are the main concerns of this technique. Previous studies have shown the incidence of these complications, especially the surgical site infection, can reach nearly 60% [7–9]. In APEF group, totally six patients developed surgical site infection, while no patient experienced this complication in modified PSRF group. The infections were controlled after being treated by intravenous antibiotic treatment for one course. In the current study, we just placed the supra-acetabular pins since it is easier to locate the dense cancellous bone at this area during the operation procedures. According to the previous studies, few surgical site infections would happen when single pins performed in the gluteus medius pillar as fewer soft tissue were traversed [12]. Nevertheless, on the basis of our clinical experience, the results would not be significantly influenced by the choice of external fixator in spite of the existence of some minor differences. Besides, APEF with external frame inevitably lead to inconvenience, to some extent, to the patients’ quality of daily life including wearing clothes, sitting, sleeping, and normal daily activities [7–9].

To manage anterior pelvic ring fractures, the minimally invasive techniques have been developed in recent years [11–15, 21]. Using pedicle screw-rod fixation to treat anterior pelvic fractures was first demonstrated by Kuttner et al. [22]. In their study, two pedicle screws were fixed in the supra-acetabular region via a curved rod connected subcutaneously. Yet, the connecting rod placed crossing the anterior inferior iliac spine (AIIS) level would make some degree of compression to the abdomen especially for the obese patients. According to our initial clinical practice using this technique, some patients were observed to have persistent pain at supra-pubic area. One possible explanation we speculated is that only two pedicle screws fixed at the AIIS, without the fixation of the pubic area, would make the pubic fracture sites unstable and result in relative micro-movement between fractured sites. Cole et al. [12, 13] performed a novel method for treating fractured anterior ring with reconstruction plate placed from the pubic symphysis to the iliac crest forming the structure of pelvic bridge to firmly fix the pelvic fracture. With the aim of combining the advantages of the pelvic bridge and pedicle screw-rod fixator, we modified the two pedicle screw-rod fixation. A third pedicle screw was fixed in either site of pubic tubercle, thus, totally three screws were employed. The rod was contoured based on the anatomy of the anterior ring and placed along the superior border of the pubis. Accordingly, with three pedicle screws fixed at pubic tubercle and AIIS respectively, a firmly three-point triangle with this fixator frame was formed which could afford more stability than the initial two-point fixator in the treatment of anterior ring fractures. By means of providing additional connection point between pedicle screws and the contoured rod, this modified fixator could better restrict the relative micro-movement between the sites of fractured pubis. More attention should be paid during the placement of the third screw at pubic area. It is worth noting that the screws neither fixed into the pubic symphysis nor closely to the lateral pubis ought to be avoided so as to protect the spermatic cord in male and round ligament in female, respectively.

No surgical site infection, loss of fixation, and loosening of implants were found in modified PSRF group compared with those in APEF group. LFCN is an easily injured tissue during the tissue dissection, placement of the rod, and the fixation removal [2]. In the current study, its irritation was observed in three patients (3/21, 14.3%) in modified PSRF group. In view of this, complication was related to the rod end length; hence, the short rod should be adopted to avoid it. Only one patient was found to experience femoral nerve palsy during the surgery. Urgent measurement was taken by adjusting the PSRF; then, the symptom was gradually relieved. The symptom eventually disappeared after PSRF being removed. Carefully surgical management and physical examination should be indispensable to prevent the occurrence of such a complication. In the light of our experience, more space should be kept between the screw and the rectus fascia.

The modified PSRF can be functioned as an effective instrument during the reduction procedure of the anterior pelvic fractures. By means of its arch structure, the reduction of open-book anterior pelvic fracture can be acquired via shortening the connected rod length, while the close-book anterior pelvic fracture can be reduced via lengthening the connected rod to regain the pelvic integrity. However, the sequence for the reduction is still controversial. Vaidya et al. [11] advocated that posterior stability should be performed as the priority. While Gardner et al. [14] demonstrated anterior fixation should be first considered for the reduction of the pelvis. On the basis of our clinical experience, taking posterior ring as the priority will be convenient for the anterior reduction and benefit for the reduction of the pelvis.

Limitations of the current study need to be stated. Firstly, this was a single-center retrospective study with relatively less samples; more cases should be taken into account to compare the application of these two methods from multi-center investigation. Secondly, the comparison between these two methods was just based on the clinical data analysis. However, the biomechanical analysis which could provide firm evidence for the conclusion should be performed. Thirdly, if another group using two-pin pedicle screw-rod fixator was added for comparison, the results would be more meaningful.

## Conclusion

In summary, both modified PSRF and APEF can afford anterior pelvic ring fracture. Compared to APEF, benefits for using modified PSRF include an easy to operate surgical technique avoiding soft tissue injuries and low incidence of nerve and vascular injuries as well as the infections of pin sites. In addition, for obese patients with anterior ring fractures, only short operative time is needed and prone position could be applied to make them comfort. The modified PSRF combining the advantages of internal fixation and the minimally invasive technique could be used as an alternative method for instable anterior pelvic fractures.

## Abbreviations

AIIS: Anterior inferior iliac spine
APEF: Anterior pelvic external fixation
LFCN: Lateral femoral cutaneous nerve
PSRF: Pedicle screw-rod fixation
